# Does the shoe-lace technique aid direct closure of fasciotomy wounds after acute compartment syndrome of the lower leg? A retrospective case-control study

**DOI:** 10.1177/14574969211019639

**Published:** 2021-06-02

**Authors:** Piia Suomalainen, Toni-Karri Pakarinen, Ilari Pajamäki, Minna K. Laitinen, Heikki-Jussi Laine, Jussi P. Repo, Ville M. Mattila

**Affiliations:** Department of Orthopaedics and Traumatology, Unit of Musculoskeletal Surgery, Tampere University Hospital, Elämänaukio, Kuntokatu 2, Tampere, 33520, Finland; Department of Orthopaedics and Traumatology, Unit of Musculoskeletal Surgery, Tampere University Hospital, Tampere, Finland; Department of Orthopaedics and Traumatology, Unit of Musculoskeletal Surgery, Tampere University Hospital, Tampere, Finland; Department of Orthopaedics and Traumatology, Helsinki University Hospital and University of Helsinki, Helsinki, Finland; Department of Orthopaedics and Traumatology, Unit of Musculoskeletal Surgery, Tampere University Hospital, Tampere, Finland; Department of Orthopaedics and Traumatology, Unit of Musculoskeletal Surgery, Tampere University Hospital, Tampere, Finland; Department of Orthopaedics and Traumatology, Unit of Musculoskeletal Surgery, Tampere University Hospital, Tampere, Finland; The School of Medicine, Tampere University, Tampere, Finland

**Keywords:** Fasciotomy, tibial fractures, compartment syndrome, tibia, fracture, shoe-lace

## Abstract

**Background and objective:**

Tibia fractures are relatively common injuries that are accompanied with acute compartment syndrome in approximately 2% to 20% of cases. Although the shoe-lace technique, where vessel loops are threaded in a crisscross fashion and tightened daily, has been widely used, no studies have compared the shoe-lace technique with the conventional one. The aim of this study was to compare the shoe-lace technique with the conventional technique.

**Methods:**

We identified 359 consecutive patients with intramedullary nailed tibia fracture and complete medical records including outpatient data between April 2007 and April 2015 from electronic patient database of our institute. The use of the shoe-lace technique was compared to conventional one (in which wounds were first left open with moist dressings). Main outcome measurement is direct closure of fasciotomy wounds.

**Results:**

From 359 consecutive patients with intramedullary nailed tibia fracture, fasciotomy was performed on 68 (19%) patients. Of these, the shoe-lace technique was used in 47 (69%) patients while in 21 (31%) patients, the shoe-lace technique was not applied. Side-to-side approximation was successful in 36 patients (77%) in the shoe-lace+ group and 7 patients (33%) in the shoe-lace– group (p = 0.002).

**Conclusions:**

The main finding of our comparative study was that the shoe-lace technique seems to ease direct closure of lower leg fasciotomy wounds, and thus reduces the frequency of free skin grafts. Our finding needs to be confirmed in a high-quality randomized controlled trial.

## Introduction

The Finnish Care Register for Health Care shows that, in Finland, approximately 1200 tibia fractures are operatively treated annually (incidence 23/100,000 person-years). Based on the literature, tibia shaft fracture is associated with acute compartment syndrome (ACS) in approximately 2% to 20% of cases.^1–5^ The risk factors for ACS can be divided to fracture-related and host-related. Host-related risk factors have been shown to be male gender, age below 55 years, arterial injury in the same leg and an Injury Severity Score (ISS) greater than 16^2,6–8^, and a diaphyseal fracture location as fracture-related risk.^7^

The symptoms of ACS include severe pain, which is more severe than what would be expected from the injury itself, using or stretching the involved muscles increase the pain and the muscle might feel tight or full. Pain is followed by nerve palsy distal to the knee and eventually a markedly compromised arterial blood supply below the knee.^9^ The gold standard for the treatment of ACS of the lower leg is immediate fasciotomy of all four muscle compartments, performed during the initial stabilization of the fracture.^10,11^

There have only been a few studies concerning the closure of fasciotomy wounds. Several dynamic dermatotraction devices and techniques have been previously introduced such as the External Tissue Extension and shoe-lace technique.^12,13^ The shoe-lace technique, in which vessel loops are threaded in a crisscross fashion and tightened bed side in the ward, was originally described by Cohn et al.^14^ in 1986. Since then, the technique has been widely used. There are, however, no published studies that compare this technique with the conventional one, where fasciotomy wounds are left open with moist dressings.

The primary aim of our study was to compare the shoe-lace technique with the conventional technique in closing lower leg fasciotomy wounds in patients with tibia fracture treated with intramedullary nailing in terms of the proportion of free skin grafts and infection rate. We also assessed whether the choice of wound closure technique had an effect on patient recovery or the complications associated with fractures.

## Patients and methods

**Fig. 1.**
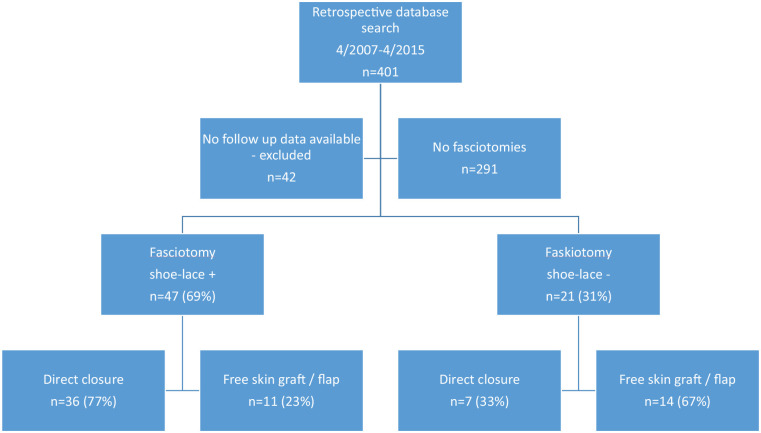
Flow chart of the study.

Our study was a retrospective consecutive patient series conducted in a Level I trauma center in Finland. Tampere University Hospital is a tertiary referral trauma center serving a catchment area of 1.2 million people. All patients with tibia fractures, as defined by the International Statistical Classification of Diseases (ICD-10) codes S82.1, S82.2, or S82.3, treated with intramedullary nailing between April 2007 and April 2015 were included in the study. Eligible patients were identified using a computer-based search of the hospital’s electronic patient records. Patients with open growth plates and patients who had their postoperative follow-up in another hospital were excluded from the study (Fig. 1).

Patient records were reviewed and patient demographics, injury mechanism and energy, date of the injury, fracture pattern, date(s) of operation(s), time to definitive fracture fixation and fasciotomy closure technique, infections, and revision surgery date(s) were all recorded. Traffic collisions and falls from heights greater than 2 meters were classified as high-energy injuries and others as low-energy injuries (Table 1).

**Table 1.** Characteristics of fasciotomy patients by treatment group (shoe-lace+/–).

	Shoe-lace–, n = 21 (31%)	Shoe-lace+, n = 47 (69%)	Significance
Sex			
Female	3 (14%)	7 (15%)	p = 0.631
Male	18 (86%)	40 (85%)	
Age			
14–20	7 (33%)	10 (21%)	p = 0.305
21–45	4 (19%)	20 (42%)	
46–60	8 (38%)	14 (30%)	
61–>	2 (10%)	3 (6%)	
Open fracture			
No	11 (51%)	36 (77%)	p = 0.057
G I	2 (10%)	4 (8%)	
G II	0 (0%)	3 (6%)	
G III A	4 (19%)	1 (2%)	
G III B	2 (10%)	2 (4%)	
G III C	2 (10%)	1 (2%)	
Energy of the injury			
Low	4 (19%)	25 (53%)	p = 0.009
High	17 (81%)	22 (47%)	
Deep infection			
Yes	2 (29%)	5 (71%)	p = 0.889
No	19 (31%)	42 (69%)	
Direct closure			
Yes	7 (16%)	36 (84%)	p = 0.001
No	14 (56%)	11 (44%)	
Time to closure, days, mean (SD; range)			
	6.6 (3.4; 1–18)	5.9 (2.5; 2–19)	p = 0.143
Hospital stay, days, mean (SD; range)			
	15.6 (19.9; 6–43)	13.7 (10.4; 5–67)	p = 0.467

SD: standard deviation.

The primary care of the patients followed the algorithm principles of the Advanced Trauma Life Support (ATLS) guidelines.^15^ Closed fractures (without compartment syndrome) were operated within 24 h after admission, whereas open fractures and fractures with comparment syndrome were operated as soon as possible (usually within 6 h, intial fracture fixation, and debridement/fasciotomy). The open fractures were classified according to the Gustilo and Anderson system (GI, GII, GIIIA, GIIIB, and GIIIC) and the fracture types according to the OTA/AO classification.^16–18^

The treatment protocol in our hospital has been to treat displaced tibia shaft fractures as well as the majority of distal extra-articular (greater than 5 cm proximal to the distal tibial plafond) metaphyseal tibia fractures with intramedullary nailing. Fasciotomy is performed in a standardized manner using a two incision technique to open four compartments with long skin incisions as soon as the clinical suspicion of compartment syndrome (i.e. tense muscle compartments, abnormal pain in the calf, painful passive calf muscle stretching, and/or sensory disturbances) is confirmed. Continuous compartment pressure measurement is addressed to those patients with an altered level of consciousness (i.e. head trauma or sedation) and in cases where compartment syndrome cannot be reliably excluded by clinical examination. After the procedure, fasciotomy wounds are left open with moist dressings.

**Fig. 2.**
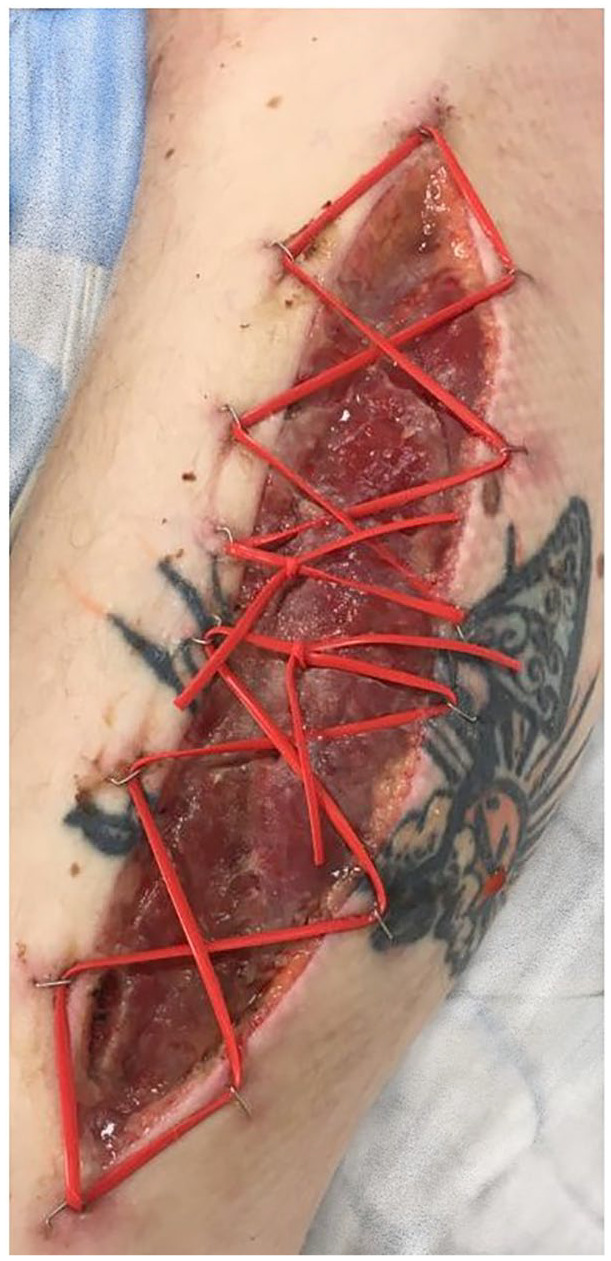
Postoperative image 3 days after surgery of shoe-lace technique used in a fasciotomy wound.

The use of the shoe-lace technique used in this study was determined by the senior consultant orthopedic trauma surgeons according to their own preferences. In the shoe-lace technique, an elastic band for vascular surgery is attached to the skin with metal clips, which were applied with surgical stapler 0.5 cm from the incision edges, starting at the proximal apex and continuing toward the distal vertex. The wire was attached to one side and passed through the incision to be attached on the opposite side, in a sequence that resembles a zigzag from the proximal to the distal regions (Fig. 2). Thereafter, the wounds are covered with moist dressings. The method was used both to medial and lateral wounds. The wounds were monitored, bed-side dressings changed, and shoe-laces (if used) tightened daily. Surgical debridement was rarely done, and decision was made case-by-case. Both fasciotomy wounds were closed at the same time as soon as the soft tissue oedema allowed, and the underlying muscle was deemed viable.

In statistical analysis, two-way tables were used with chi-square and Fisher’s test. Significance level was set to p < 0.05. The t-test was used when we analyzed continuous, unskewed data, whereas the Mann–Whitney U test was used for continuous, skewed data. We used SPSS v.23.0 for statistical analysis. The manuscript has been prepared using STROBE checklist.

## Results

In total, 401 tibia fractures were treated with intramedullary nailing at our institute between April 2007 and April 2015. We excluded 42 patients due to insufficient outpatient clinic data, and thus the final study sample comprised 359 patients. The mean age of the patients was 44 years (range from 14 to 98 years) and there were 110 women (31%). Most of the patients (n = 348, 97%) were treated with definitive intramedullary fixation within 24 h after admission, while 11 patients underwent external fixation as the primary treatment and intramedullary nailing later. The majority of the fractures were located in the mid- or distal shaft (n = 292, 81%). In addition, 62 fractures (17%) treated with intramedullary nailing were located in the distal metaphysis.

There were 67 (19%) open fractures (grade I n = 26, 39%, grade II n = 11, 16%, grade IIIA n = 13, 19%, grade IIIB n = 14, 21%, grade IIIC n = 4, 6%). The highest proportion of open fractures was seen in young men (aged 14–20 years) resulting from high-energy injuries. Furthermore, this group had the highest infection rate (12%).

Fasciotomy was performed on 68 (19%) patients in our sample (Table 1). The shoe-lace technique was used on 47 (69%) patients (shoe-lace+ group) while in 21 (31%) patients, shoe-laces were not applied (shoe-lace– group). Data analysis showed that the shoe-lace+ and shoe-lace– groups were comparable in all but one category; the shoe-lace– group had a statistically lower proportion of low-energy injuries (n = 4, 19%) than the shoe-lace+ group (n = 25, 53%) (p = 0.009) (Table 1).

Side-to-side approximation was successful in 36 patients (77%) in the shoe-lace+ group and in 7 patients (33%) in the shoe-lace– group (p = 0.002) (Table 1). Direct closure was not possible for 23% of patients in the shoe-lace group as the volume of the muscle was not constrained by the fascia leading to greater surface area to be covered. The majority of the reoperations for secondary closures were done with a free skin graft (n = 17, 68%), and the others needed flap reconstruction mainly due to severe soft tissue defects (latissimus dorsi free flap, n = 4, 16%, anterolateral thigh free flap, n = 1, 4%, soleus local flap, n = 2, 8%, peroneus brevis local flap, n = 1, 4%) (Fig. 1).

The overall deep infection rate was 6% (23/359) in the whole study sample (with or without fasciotomy), and there was no statistical difference in deep infections (without fasciotomy 16/291), 5%, with fasciotomy (7/68), 10% (p = 0.109). In addition, the timing of the closure was not associated with deep infection rate (p = 0.355). However, patients who underwent the free skin graft/flap method to cover the fasciotomy wounds had a higher risk for infections when compared to patients with direct closure (p = 0.045).

Reoperations were performed on 14 patients (4%) because of delayed fracture healing and on 14 patients (4%) because of flap or skin graft problems (Table 2).

**Table 2.** Reoperations and their indications in the study population with tibia fractures treated with intramedullary nailing by fasciotomy status (n = 359).

Operation	Indication for operation	Fasciotomy–, n = 291 (%)	Fasciotomy+, n = 68 (%)
Removal of the nail			
	Infection	8 (3%)	5 (7%)
	Mechanical symptom	8 (3%)	1 (1%)
Soft tissue operation	Problems with flap/free skin graft	7 (2%)	8 (12%)
Exchange of the nail/other hardware operation	Non-union of the fracture	11 (4%)	3 (4%)

## Discussion

The main finding of our comparative study was that the shoe-lace technique seems to ease direct closure of lower leg fasciotomy wounds, and thus reduces the need for free skin graft. To the best of our knowledge, this is the first study to compare the shoe-lace technique with conventional fasciotomy wound treatment with moist dressing. Compartment syndrome of the lower leg is associated with tibia fractures in approximately 2% to 20% of cases.^1–5,19,20^ This finding is in line with our own results, as fasciotomy was performed on 69 patients (19%) who had undergone tibia fracture nailing.

Shoe-lace technique in comparison to vacuum-assisted closure (VAC) has been studied in few publications in a randomized controlled trial setting. Both shoe-lace technique and VAC have been found effective, safe, and reliable methods. Shoe-lace technique, in accordance to our results, has been found to have a reduction of skin grafts in comparison to VAC.^21^ Compared to the shoe-lace technique, however, negative pressure therapy takes longer until definite skin closure and is far more expensive. Hospitalization time and cost of treatment can be significantly increased should additional skin grafting be required.^13^

It has been suggested that the closure of fasciotomy wounds should be done as soon as possible provided there is no longer swelling of the calf muscles.^13^ In our sample, fasciotomy wounds were closed approximately 6 days postoperatively with either direct closure, a free skin graft, or a plastic surgical flap. Weaver et al.^22^ found in their study that if the direct closure of the wounds was not possible in the first revision operation, it was highly unlikely to succeed at all, and therefore free skin graft should be considered.

Direct closure of the fasciotomy wounds was achieved with 44 (65%) of the patients in our study. Majority of these patients had the closure done through the shoe-lace technique (n = 36, 77%). Although this technique had already been described in 1986 by Cohn et al.^14^, there have been no previously published studies that have compared the shoe-lace technique with the conventional method where the wounds are left open without any tensioning aid. The shoe-lace technique seems to also diminish the need for free skin grafts, which is a statistically and clinically significant finding. The number of open fracture patients was comparable since in both groups with 10 open fracture patients in each group. There were 26% more patients with closed fracture in the group of shoe-lace closure. However, the shoe-lace– group had more high-energy injuries. Patients in the shoe-lace– group could potentially have less direct closures due to the fact that they had higher number of high-energy fractures potentially leading to more extensive soft tissue damage. There was no statistically significant difference in hospital stay between the two groups. A longer hospital stay in patients without shoelace technique could have been expected. However, the hospital stay may also be influenced by other factors, such as a high-energy injury, which was more common in the shoe-lace– group.

The strengths of the study include the standardized care of the tibia fracture patients and the fact that we included all patients with tibia fracture and intramedullary nailing. The weaknesses are the retrospective and comparative study design. Although the demographics of the patients in both groups were similar, it is possible that a selection bias exists, since the decision to use the shoe-lace technique was based on a surgeon’s own opinion. Moreover, in the shoe-lace– group, high-energy traumas were overrepresented. Also, as the group sizes were relatively small, the statistical analyses could be prone to type II error. In this study, we did not investigate the cosmetic result due to the retrospective nature of the study design.

Reoperations were done in 4% of the cases, and infection was the most common reason for tibia nail removal. Although the infection rate in our material was rather low (6%), a high infection rate was observed in young men, most commonly after high-energy injuries and open fractures. This finding is in accordance with the findings of Papakostidis et al. who reported an association between severe open fractures and infections. They reported that Gustilo Gr III fractures are associated with more complications than the more benign injuries.^23^

The subgroup of young men was overrepresented in the fasciotomy group. This is justified by evidence that shows the risk factors for compartment syndrome are typically young age, male gender, and high-energy injury.^1^ Another risk factor for compartment syndrome is Gustilo Gr III open fracture, which causes not only soft tissue defect but often gives indication of high-energy injury. This can also be seen in our material, as almost half of the patients with Gustilo Gr III open fracture needed fasciotomy, while the corresponding figure in patients with closed fractures was only 16%.

## Conclusion

The conclusion of this study is that the shoe-lace method seems to aid direct closure of fasciotomy wounds after ACS of the lower leg. However, this finding needs to be confirmed in a high-quality randomized controlled trial.
